# Expression patterns of angiogenic and lymphangiogenic factors in ductal breast carcinoma *in situ*

**DOI:** 10.1038/sj.bjc.6602567

**Published:** 2005-04-19

**Authors:** P Wülfing, C Kersting, H Buerger, B Mattsson, R Mesters, C Gustmann, B Hinrichs, J Tio, W Böcker, L Kiesel

**Affiliations:** 1 Department of Obstetrics and Gynecology, University of Münster, Albert-Schweitzer-Str. 33, 48129 Münster, Germany; 2 Gerhard-Domagk-Institute of Pathology, University of Münster, Albert-Schweitzer-Str. 33, 48129 Münster, Germany; 3 Department of Hematology and Oncology, University of Münster, Albert-Schweitzer-Str. 33, 48129 Münster, Germany; 4 Institut für Pathologie, St Vincenz-Krankenhaus Limburg, Germany; 5 Institut für Pathologie, Köln-Rodenkirchen, Germany

**Keywords:** angiogenesis, lymphangiogenesis, immunohistochemistry, breast cancer, ductal carcinoma *in situ*, growth factors, receptors

## Abstract

The objective of this study was to investigate expression of various growth factors associated with angiogenesis and lymphangiogenesis and of their receptors in ductal carcinomas *in situ* of the breast (DCIS). We studied protein expression of basic fibroblast growth factor (bFGF), vascular endothelial growth factor (VEGF)-A, endothelin (ET)-1, and VEGF-C, and their receptors bFGF-R1, Flt-1, KDR, ET_A_R, ET_B_R, and Flt-4 immunohistochemically in 200 DCIS (*pure* DCIS: *n*=96; DCIS *adjacent* to an invasive component: *n*=104) using self-constructed tissue microarrays. Basic fibroblast growth factor-R1, VEGF-C, Flt-4, and ET_A_R were expressed in the tumour cells in the majority of cases, whereas bFGF and Flt-1 expression was rarely observed. VEGF-A, KDR, ET-1, and ET_B_R were variably expressed. The findings of VEGF-C and its receptor Flt-4 as lymphangiogenic factors being expressed in tumour cells of nearly all DCIS lesions and the observed expression of various angiogenic growth factors in most DCIS suggest that *in situ* carcinomas are capable of inducing angiogenesis and lymphangiogenesis. Moreover, we found a higher angiogenic activity in pure DCIS as compared to DCIS with concomitant invasive carcinoma. This association of angiogenic factors with pure DCIS was considerably more pronounced in the subgroup of non-high-grade DCIS (*n*=103) as compared with high-grade DCIS (*n*=94). Determination of these angiogenic markers may therefore facilitate discrimination between biologically different subgroups of DCIS and could help to identify a particularly angiogenic subset with a potentially higher probability of recurrence or of progression to invasiveness. For these DCIS, targeting angiogenesis may represent a feasible therapeutic approach for prevention of progression of DCIS to invasion.

Angiogenesis is known to be a prerequisite for tumour growth beyond a few mm^3^ in size (Folkman, 1995). Several studies have shown that the angiogenic potential as assessed by tumour microvessel density (MVD) of breast carcinomas correlates with tumour progression and metastasis, thus predicting a poor clinical outcome in breast cancer patients (Gasparini *et al*, 1994; Heimann *et al*, 1996). Also, lymphangiogenesis in breast cancer is supposed to contribute to tumour progression and poor survival by predisposing to metastatic spread via the lymphatic system (Nakamura *et al*, 2003).

The family of vascular endothelial growth factor (VEGF) proteins is a group of angiogenic factors that regulate the growth of endothelial cells. There are four major VEGF isoforms, VEGF-A, VEGF-B, VEGF-C, and VEGF-D. Three main tyrosine-kinase receptors (RTKs) have been identified: VEGFR-1 (also known as Flt-1) (de Vries *et al*, 1992), VEGFR-2 (also known as Flk-1 in mouse or KDR in human) (Millauer *et al*, 1993), and VEGFR-3 (also known as Flt-4) (Pajusola *et al*, 1994).

Vascular endothelial growth factor-A is considered to be the most crucial regulator of angiogenesis and vasculogenesis (Ferrara *et al*, 2003) and its expression has been demonstrated in cancer cells of various human tumours including breast cancer (Yoshiji *et al*, 1996; De Jong *et al*, 1998a). In breast carcinomas, VEGF-A expression correlates with angiogenesis and seems to represent a useful prognostic marker for poor outcome (Obermaier *et al*, 1997; Gasparini, 2001). The biological effects of VEGF-A are mediated by Flt-1 and KDR binding VEGF-A with high affinity (Mustonen and Alitalo 1995). Although Flt-1 was the first receptor to be identified as a VEGF receptor (de Vries *et al*, 1992), there is much evidence that Flt-1 performs an inhibitory role in mitogenesis of endothelial cells by sequestering VEGF-A and preventing its interaction with KDR, whereas KDR is considered to be the major mediator of angiogenesis (Ferrara *et al*, 2003). Initially, expression of Flt-1 and KDR was believed to be restricted to the vascular endothelium, but to date these receptors have also been detected in several types of nonendothelial cells including breast cancer cells, suggesting an autocrine effect of VEGF-A on tumour cells (De Jong *et al*, 1998a; Kranz *et al*, 1999). The clinical significance of VEGF-A receptor expression in breast cancer is yet unclear. On the one hand, it has been reported that Flt-1 overexpression improves survival in breast cancer, whereas KDR expression is associated with a poor prognosis (Zhukova *et al*, 2003). However, another study has demonstrated, Flt-1 to be related to a higher metastatic and local recurrence risk in breast cancer patients (Dales *et al*, 2004).

Flt-4 is an RTK that is similar to the two VEGF-A receptors in structure but primarily represents a marker for lymphatic endothelial cells and which is also expressed in epithelial cells of various malignant tissues. Although VEGF-C appears to be the primary lymphangiogenic factor, it also links angiogenesis with lymphangiogenesis, as it can stimulate both processes by activating Flt-4 (Jeltsch *et al*, 1997) and KDR (Cao *et al*, 1998), respectively. Moreover, an autocrine growth stimulation pattern of VEGF-C via Flt-4 has been suggested in tumours (Van Trappen *et al*, 2003). In breast cancer, expression of VEGF-C and Flt-4 is associated with angiogenesis and lymphangiogenesis (Valtola *et al*, 1999; Kinoshita *et al*, 2001). Also, a clinicopathological significance of VEGF-C and Flt-4 in breast cancer has been described, since their expression correlated with lymph node metastasis and decreased survival of patients (Kinoshita *et al*, 2001; Nakamura *et al*, 2003).

Another category of angiogenic activators evaluated in human breast carcinomas is the fibroblast growth factor (FGF) family. FGFs are important signalling molecules contributing to angiogenesis, tumour growth, and tumour progression (Mason, 1994; Kern and Lippman, 1996). Among FGFs, basic FGF (bFGF) which binds to specific RTKs, FGFRs (Mohammadi *et al*, 1997), is the most active growth factor for endothelium and several studies have demonstrated its expression in breast cancer (Luqmani *et al*, 1992; Colomer *et al*, 1997; Smith *et al*, 1999). However, the clinical relevance of bFGF expression in breast cancer is yet unclear, since conflicting results have been reported (Colomer *et al*, 1997; Smith *et al*, 1999).

Recently, the endothelin (ET)-axis comprising the vasoactive peptide ET-1 and the cell-surface receptors, ET_A_R and ET_B_R, came into focus due to its emerging role in cancer (Nelson *et al*, 2003). Increased ET-1 expression has been demonstrated in various human malignancies as well as in breast cancer. It appears that the effects of the ET-axis on tumorigenesis and tumour progression are mediated by several mechanisms including proliferation, angiogenesis, and inhibition of apoptosis (Nelson *et al*, 2003). Endothelin-1 contributes to the process of angiogenesis, stimulating endothelial cell growth predominantly through ET_B_R and inducing vascular smooth muscle cell and pericyte mitogenesis mediated through ET_A_R (Bek and McMillen, 2000; Salani *et al*, 2000). Previously, we have demonstrated an increased expression of the ET-axis in breast carcinomas. Expression of ET_A_R correlated with aggressive types of breast cancer and poor clinical outcome, indicating a potential connection between the ET-axis and disease progression in breast cancer (Wülfing *et al*, 2003a). Similar to findings in ovarian cancer (Salani *et al*, 2000), we observed a significant correlation between expression of the ET axis and neovascularisation and VEGF expression of breast carcinomas (Wülfing *et al*, 2004b). Further studies have suggested that activation of ET_A_R by ET-1 induces the production of VEGF, which in turn stimulates tumour growth and angiogenesis by increasing the levels of hypoxia-inducible factor-1 (HIF-1*α*), in a time- and dose-dependent manner (Spinella *et al*, 2002).

The switch of a tumour to an angiogenic phenotype is considered to be critical for progression and metastasis (Folkman *et al*, 1989). In DCIS, two distinct vascular patterns have been described: (1) an increased stromal vascular density which is thought to result from angiogenic factors being released by accessory cells; (2) an increase in periductal vessels as an effect of angiogenic factors secreted by intraductal tumour cells (Guidi *et al*, 1994; Engels *et al*, 1997).

This last pattern has been shown to correlate with the development of an invasive recurrence (Teo *et al*, 2003). Thus, it is conceivable that an increase in periductal angiogenesis may contribute to the transformation from DCIS to invasive carcinoma. In the present study, we therefore analysed expression of several surrogate markers of angiogenesis and lymphangiogenesis in DCIS using a tissue microarray (TMA) with 902 cores from 200 DCIS specimens. The objective was to see whether these markers are associated with established histopathological characteristics and to assess whether there are different expression patterns between the groups of DCIS with (*n*=104) and without (*n*=96) coexistent invasive carcinoma.

## MATERIALS AND METHODS

### Patients and tumours

A total of 200 patients with DCIS were included in the study. Among these, 96 patients had a *pure* DCIS and in 104 patients the DCIS was associated with an invasive breast carcinoma (*coexistent* DCIS). All cases were classified according to the criteria outlined by Holland *et al* (1994) considering the nuclear grading and architectural features. Based on this classification, cases were graduated as low grade (*n*=54), intermediate grade (*n*=49), and high grade (*n*=94). With respect to these criteria, cases were divided into ‘non-high-grade’ (low grade + intermediate grade) or ‘high-grade’ DCIS. The median age of patients was 59 years (range 18–94 years).

### Breast cancer TMA

**Figure 1:**
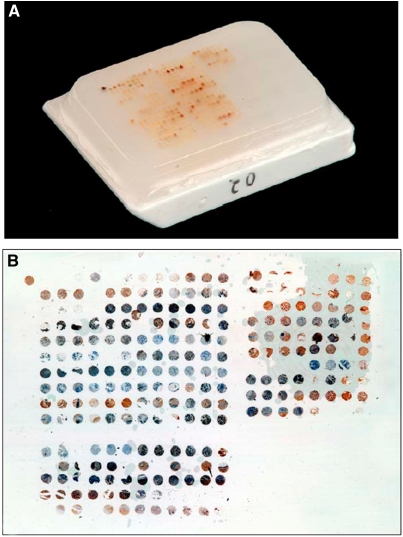
Tissue microarray. (**A**) Picture of a TMA; (**B**) stained section of a TMA.

Routinely fixed paraffin-embedded tissue blocks containing DCIS were extracted from the files of pathology laboratories serving as donor blocks for the TMA. Sections were cut from each donor block and stained with haematoxylin and eosin. Using these slides morphologically representative regions were chosen from each of the 200 tumour samples and circled. At least four cylindrical 0.6 mm cores were acquired from the circled areas of each DCIS sample and precisely arrayed into a new recipient paraffin block (20 × 35 mm) using a manual tissue arrayer (Beecher Instruments, Silver Spring, MD, USA). The final tissue set consisted of four blocks each containing 116–306 tumour sample cores for a total of 902 cores (Figure 1). The presence of DCIS in the arrayed samples was verified on haematoxylin–eosin-stained sections.

### Immunohistochemistry

Table 1 presents all antibodies, dilutions, incubation times, and antigen-retrieval methods used. Tissue microarray blocks were cut with a microtome into 4 *μ*m sections that were mounted on poly-L-lysine-coated glass slides and processed for immunohistochemistry. After deparaffinisation and rehydration, unspecific binding was blocked. Then, antigen retrieval by different pretreatment methods was followed by incubation with the primary antibody. After detection, sections were counterstained with haematoxylin. Appropriate negative (the first antibody was either omitted or replaced by non-immune rabbit IgG diluted to the same concentration as the first antibody) and positive (as shown in Table 1) controls were used throughout. Additionally, DCIS-TMA sections were stained for ER, PR and Her-2/neu by standard immunohistochemical methods as described previously (Wülfing *et al*, 2003b).

### Quantification

Semiquantitative analysis of staining results from 902 tissue array cores was performed by two investigators in a blind-trial fashion without knowing the histopathological data for the corresponding case. Depending on the staining procedure varying numbers of tissue cores were detached, others did not contain a sufficient number of tumour cells. Also, if precise diagnosis of DCIS on the TMA section was not possible, the case was disregarded. Therefore, some cases could not be analysed.

For evaluation of bFGF-R1, ET-1, ET_A_R, and ET_B_R expression, intensity of the cytoplasmic immunostaining was scored semiquantitatively on a four-tiered scale (negative=0, weak=1+, moderate=2+, strong=3+). We defined samples with a moderate (2+) or strong (3+) cytoplasmic immunostaining intensity to have an elevated expression of this marker and thus to be ‘positive’, respectively (Wülfing *et al*, 2004b). A similar score was used for VEGF-C and Flt-4, both showing a cytoplasmic and nuclear immunostaining, and for KDR expression, which was primarily observed in the cytoplasm but additionally showed a staining of the cell membranes. For Flt-1, the cytoplasmic immunostaining intensity was classified from 0 (negative) to 2+ (moderate). Cases scored as ⩾1+ were considered to be Flt-1 positive. Expression of bFGF and VEGF-A was characterised as a negative or positive reaction according to both the intensity of the immunostaining and the percentage of stained tumour cells. Samples were judged to be positive if ⩾10% of the tumour cells showed moderate or strong cytoplasmic immunoreaction (De Jong *et al*, 1998a). Her-2/neu staining was scored 0 (absent) to 3+ (maximum cytomembranous staining), with a score ⩾2+ considered Her-2/neu-positive. ER and PR scores were calculated as the percentage of positively stained nuclei. ER and PR status were defined positive when ⩾10% nuclei stained positively.

### Data analysis

Staining results for the angiogenic and lymphangiogenic markers were correlated with results for ER, PR, and Her-2/neu, as well as with the nuclear grading of DCIS. According to the clinical practice, low-grade and intermediate-grade cases were taken together as ‘non-high-grade’ as opposed to the poorly differentiated ‘high-grade’ DCIS. Correlations were tested for statistical significance by cross-tables, applying Pearson's *χ*^2^ test and Fishers' exact test (SPSS 10.0). Differences between the groups of DCIS with *vs* DCIS without an invasive carcinoma, as well as correlations between coexistent DCIS and the respective invasive component were also tested using *χ*^2^ test.

## RESULTS

### Immunohistochemistry of the DCIS specimens

Detailed staining results are depicted in Table 2. In the majority of cases, tumour cells of DCIS showed expression of Flt-4, bFGF-R1, VEGF-C, and ET_A_R, whereas Flt-1 and bFGF expression was rarely observed. A positive immunoreaction for KDR, ET-1, VEGF-A, and ET_B_R in the tumour cells was present in a varying number of samples. The following immunohistochemical staining patterns of DCIS were observed:

### Vascular endothelial growth factor-A and VEGF-receptors 1 (Flt-1) and 2 (KDR)

**Figure 2:**
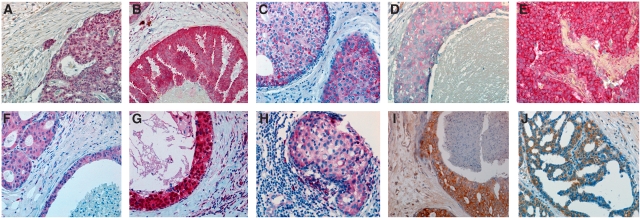
Ductal carcinoma *in situ* specimens with representative immunohistochemical staining patterns for (upper row) bFGF (**A**), bFGF-R1 (**B**), VEGF-A (**C**), Flt-1 (**D**), KDR (**E**), and (lower row) VEGF-C (**F**), Flt-4 (**G**), ET-1 (**H**), ET_A_R (**I**), ET_B_R (**J**).

*Vascular endothelial growth factor-A:* A positive immunoreaction for VEGF-A was observed in about half of the DCIS samples. Most cases presented with a faint or moderate cytoplasmic staining of tumour cells (Figure 2C). Normal glandular cells were negative for VEGF-A, while myoepithelial cells stained positively. The stromal and connective tissue was mostly negative for VEGF-A.

*Flt-1:* Expression of Flt-1 in the tumour cells was found in very few cases of DCIS (16.1%). Flt-1-positive cells showed a finely granular staining of the cytoplasm (Figure 2D). The peritumoral stroma often stained positively in Flt-1 positive cases. Normal glandular cells were Flt-1-negative.

*KDR:* About half of the DCIS cases (53.5%) were considered to be KDR-positive. KDR features a strong cytoplasmic staining (Figure 2E). Normal glandular cells usually stained positively, while stroma and connective tissue remained negative.

### Vascular endothelial growth factor-C and VEGF-receptor 3 (Flt-4)

*Vascular endothelial growth factor-C:* In all, 88% of DCIS showed intense staining and were considered to be VEGF-C-positive. Expression of VEGF-C protein was observed in the cytoplasm of tumour cells (Figure 2F). Little or no staining for VEGF-C was observed in normal breast epithelium. Stromal and connective tissues were VEGF-C negative.

*Flt-4:* For Flt-4, a strong staining of tumour cells was observed in most cases (95.4%) (Figure 2G). Also, normal glandular cells were often positive, while stromal and connective tissues were Flt-4-negative.

### Basic fibroblast growth factor and bFGF receptor

*Basic fibroblast growth factor:* Positive nuclear staining of tumour cells with bFGF was present in only a few cases (12.3%). In a small proportion of these positive cases, also a faint cytoplasmic staining was observed (Figure 2A). Normal glandular cells often showed immunoreaction for bFGF, while the stroma generally was bFGF-negative.

*Basic fibroblast growth factor-R1:* Almost all DCIS (94.4%) showed a strong and homogenous cytoplasmic expression of bFGF-R1 in the tumour cells (Figure 2B); in few cases, also a nuclear staining was observed. In bFGF-R1-positive cases, stromal cells often exhibited a faint staining, too, while normal glandular cells generally were moderately positive.

### Endothelin-axis

Positive cytoplasmic staining of tumour cells was observed for ET-1 in 48.4%, for ET_A_R in 76.4%, and for ET_B_R in 37.7% of cases (Figure 2 H–J). Normal glandular cells were mostly negative; in ET-positive cases, occasionally a weak staining of stromal components was present.

### Autocrine loops

Expression patterns in DCIS were screened for cases in which the receptor and ligand are both expressed in the same cells, suggesting potential autocrine loops. Possible autocrine loops were observed for all receptor/ligand combinations in varying numbers of cases (Table 3). Coexpression of ligand and receptor in the tumour epithelium was most frequently found for the VEGF-C/Flt-4 combination (87% of cases), followed by the combinations of VEGF-C/KDR (51%), ET-1/ET_A_R (37%), ET-1/ET_B_R (26%), and VEGF-A/KDR (25%).

### Association of angiogenic markers with histopathological features

The following associations between expression of angiogenic markers and other histopathological parameters were observed: ET_A_R and ET_B_R expression correlated with ER-negative DCIS (*P*=0.017 and *P*<0.001, respectively). In contrast, bFGF expression was associated with ER expression (*P*=0.080), PR expression (*P*=0.003), lack of Her-2/neu expression (*P*=0.013), and with non-high-grade DCIS (*P*=0.005). For Flt-1, a trend towards a negative correlation with Her-2/neu expression was observed (*P*=0.097). No further associations between angiogenic markers and histopathological features examined were found.

### Different expression patterns in pure DCIS *vs* DCIS with a coexisting invasive carcinoma

Comparing the expression of angiogenic markers between the groups of DCIS with (*n*=104) *vs* DCIS without (*n*=96) coexisting invasive carcinoma revealed that expression of VEGF-C, KDR, Flt-4, and ET_A_R was significantly more frequent in *pure* DCIS than in DCIS with an adjacent invasive carcinoma (Table 2). These differences between both DCIS groups were significantly more pronounced in the subgroup of non-high-grade DCIS as compared with high-grade DCIS (Table 4). In contrast, Flt-1 was significantly higher expressed in the group of DCIS with an adjacent invasive carcinoma irrespective of nuclear grading (Tables 2 and 4).

To evaluate whether these different expression patterns of angiogenic markers between pure and coexistent DCIS merely reflect an unequal distribution of DCIS with respect to histopathological and clinical features between both groups, possible associations with nuclear grading, ER-, PR-, and Her-2/neu-status and patients' age were analysed. As shown in Table 5, no significant differences were observed between both groups with respect to these parameters.

### Correlations between *in situ* and invasive carcinoma

To assess whether expression of classic histopathological factors and angiogenic markers in coexistent DCIS is determined by expression patterns in the respective invasive carcinomas, staining results for the *in situ* and the invasive (IBC) components of each patient in the coexistent DCIS group were compared. Among the angiogenic factors, we focused on those which were differentially expressed in the groups of pure and coexistent DCIS. Analysis of ET_A_R (ET_A_R-positive; DCIS: 67.9% *vs* IBC: 65.5% of cases), VEGF-C (VEGF-C positive; DCIS: 80.4% *vs* IBC: 88.6%), Flt-4 (Flt-4 positive; DCIS: 92.3% *vs* IBC: 93.3%), and Her-2/neu (Her-2/neu positive; DCIS: 21.9% *vs* IBC: 16.4%) expression revealed a similar frequency of positive staining in the *in situ* and the invasive carcinomas. In contrast, expression of ER (ER-positive; DCIS: 26.9% *vs* IBC: 61.5% of cases), PR (PR-positive; DCIS: 35.1% *vs* IBC: 48.1%), Flt-1 (Flt-1 positive; DCIS: 28.1% *vs* IBC: 44.2%), and KDR (KDR positive; DCIS: 45.1% *vs* IBC: 59.6%) was significantly more frequent in the invasive component than in the coexistent *in situ* lesion (each *P*<0.05). Moreover, a close concordance was observed with respect to nuclear grading of the *in situ* and histological grading of the respective invasive component (Kruskal Wallis test; *P*<0.001).

## DISCUSSION

Tumour progression is characterised by the uncontrolled growth of tumour cells which may then invade the surrounding host tissue and metastasise to distant organs. The growth of small tumours is initially limited by the distance beyond which nutrients and oxygen can diffuse. Thus, angiogenesis is known to be a prerequisite for tumour growth beyond a few (1–3) mm^3^ in size. Following the ‘angiogenic switch’ of a tumour, rapid tumour growth, local invasion, and, ultimately, metastasis occur (Folkman *et al*, 1989). The identification of an increased vascular density around DCIS in a number of studies (Guidi *et al*, 1994; Engels *et al*, 1997; Teo *et al*, 2003) and the observed association of higher vascularity in DCIS with invasive recurrence (Teo *et al*, 2003) have lead to the hypothesis that an angiogenic switch in DCIS may contribute to the transformation from *in situ* to invasive carcinoma. In DCIS, two distinct vascular patterns have been described: (1) a diffuse increase of MVD in the stroma and (2) an increase in the number of periductal microvessels, the latter presumably due to the direct release of angiogenic factors by neoplastic cells within the ducts (Guidi *et al*, 1994; Engels *et al*, 1997).

Angiogenesis as a multistep process is controlled by various positive and negative regulatory signals. Endogenous proangiogenic factors that could have an impact on regulation of angiogenesis in DCIS are, for example, VEGF, bFGF, ET-1, and their respective receptors; the sole exception to this is VEGFR-1 (Flt-1) which is supposed to mediate antiangiogenic effects. In the present study, we analysed expression of several angiogenic as well as lymphangiogenic growth factors and their receptors in a large series of DCIS to evaluate potential associations with established histopathological characteristics and to assess whether there are different expression patterns between the groups of DCIS *with* (*n*=104) and *without* (*n*=96) coexistent invasive carcinoma.

Vascular endothelial growth factor-A and its receptor KDR were expressed in the tumour cells of about half the cases, whereas Flt-1 expression was rarely observed (16%). Expression of VEGF-A in tumour cells of DCIS has been reported previously and was demonstrated to correlate with the degree of angiogenesis (Guidi *et al*, 1997). However, to the best of our knowledge, the present publication is the first to describe expression of VEGF-A receptors, KDR and Flt-1, in DCIS. In general, KDR is recognised as the predominant signal transducer of tumour angiogenesis, whereas Flt-1 (and especially its soluble form) is a negative regulator of VEGF availability (Ferrara *et al*, 2003). In invasive breast cancer, expression of Flt-1 and KDR has been observed in 44 and 38% of cases, respectively (De Jong *et al*, 1998a). Our finding of a rare Flt-1 expression as opposed to a considerably higher rate of KDR expression may therefore point to a presumably increased angiogenic activity in DCIS paralleled by the downregulation of antiangiogenic receptors while the so-called angiogenic switch.

In this study, VEGF-C and Flt-4, established mediators of angiogenesis (Cao *et al*, 1998; Valtola *et al*, 1999) and in particular of lymphangiogenesis (Jeltsch *et al*, 1997; Kinoshita *et al*, 2001), were expressed in the tumour cells of the majority of DCIS specimens (88 and 95%, respectively). For invasive breast cancer, a similar rate of VEGF-C-positive cases has been reported previously (Nakamura *et al*, 2003). To date, there is only one other study published investigating VEGF-C expression in a small series of DCIS (*n*=8). In that series, VEGF-C was secreted by the intraductal carcinoma cells and was suggested to act as a growth factor for Flt-4-positive periductal blood vessels and (less evidently) for lymphatic vessels (Valtola *et al*, 1999). This implicates an association of Flt-4 and its ligand VEGF-C with angiogenesis in DCIS. However, there are no published data on Flt-4 expression in tumour cells of DCIS yet. Our findings on the presence of Flt-4 expression in the majority of intraductal tumour cells also suggest an autocrine growth-stimulatory pattern of VEGF-C via Flt-4 as described for cervical cancer (Van Trappen *et al*, 2003). However, a recently published study of Vleugel *et al* (2004) demonstrated that no intralesional increase in lymph vessel density occurs during breast carcinogenesis. Nonetheless, in general it is without doubt that the spread of tumour cells via the lymphatic system is one of the major causes of metastasis and consecutively tumour-related death. Therefore, the lymphangiogenic effects of intratumorously synthesised VEGF-C may be explained by a perilesional induction of stromal lymphangiogenesis.

In the present study, bFGF expression was rarely observed in DCIS (12%), whereas almost all DCIS cases showed bFGF-R expression. To date, there are no further data available in the literature on expression of bFGF and its receptors in DCIS. Our observation on the small proportion of bFGF-expressing DCIS specimens strongly contrasts reports on a higher rate (44%) of bFGF expression in invasive breast carcinomas (De Jong *et al*, 1998a). This may reflect an intermediate position of DCIS between normal breast tissue and invasive breast cancer, the latter reportedly showing increased bFGF levels as compared to nonmalignant tissue (Smith *et al*, 1999). Nonetheless, in DCIS, we observed an association between higher bFGF expression with favourable prognostic criteria (low grade, positive steroid hormone receptor status, and lack of Her-2/neu expression), as described earlier for invasive breast cancer (Smith *et al*, 1999). This is also in good agreement with survival data in patients with invasive breast carcinomas in which bFGF expression was found to correlate with a longer disease-free interval (Colomer *et al*, 1997). Thus, although it has been shown that bFGF expression is related to higher angiogenic activity (De Jong *et al*, 1998b), bFGF may represent an indicator for invasive breast carcinomas with favourable prognosis as well as for DCIS lesions less likely to progress to invasive breast cancer.

In our study, ET-1 was expressed in the tumour cells of 48%. However, the ET receptors, ET_A_R and ET_B_R, showed an unequal distribution: ET_A_R was present in the majority (76%) of DCIS whereas ET_B_R expression was found significantly less frequently (38%). We have previously shown that expression of the ET-axis is increased in invasive breast cancer and that, in particular, ET_A_R expression correlates with more aggressive tumour types and poor survival (Wülfing *et al*, 2003a). Moreover, we found a significant positive correlation between expression of the ET-axis and vascularity as well as VEGF expression of breast carcinomas (Wülfing *et al*, 2004b). Thus, the ET-axis and especially ET_A_R may represent a marker of malignant and angiogenic activity in breast cancer. In another study comparing expression of the ET-axis in a series (*n*=88) of invasive breast carcinomas, concomitant DCIS, and normal breast tissue, we demonstrated a stepwise increase of ET-1 and ET_A_R expression with disease progression, suggesting that expression of these proteins correlates with the acquisition of malignant potential and invasive behaviour (Wülfing *et al*, 2004a). The present study also highlights the potential role of ET_A_R expression in breast carcinogenesis. These findings may have clinical relevance since ET_A_R represents a potential target for antiangiogenic and antitumorigenic therapy (Nelson *et al*, 2003). It is therefore conceivable that the administration of selective ET_A_R antagonists may prevent transformation from intraductal to invasive breast cancer.

Ductal carcinoma *in situ* is heterogeneous in its histology and clinical presentation, and so far there is no generally accepted model to predict progression to invasive carcinoma. Pure DCIS does not have the potential to metastasise and thereby leading to death. Therefore, the particular importance of DCIS is the risk of developing to invasive carcinoma.

In this study, comparative analysis of expression of these growth factors and their respective receptors in the groups of pure DCIS *vs* coexistent DCIS revealed that expression of proangiogenic factors such as VEGF-C, KDR, Flt-4, and ET_A_R was significantly more common in *pure* DCIS than in DCIS with an adjacent invasive carcinoma (*coexistent* DCIS). Conversely, Flt-1 as a negative regulator of VEGF availability and thus being regarded as an antiangiogenic receptor was expressed significantly more frequently in the group of *coexistent* breast carcinomas. These results indicate a higher angiogenic activity in DCIS *without* coexistent invasive carcinoma (*pure* DCIS). Several explanations are feasible for this rather unexpected finding: Our data, demonstrating quantitative differences between expression of angiogenic factors, suggest a biological difference among the groups of (a) pure DCIS and (b) DCIS with concomitant invasive carcinoma. This is consistent with findings from Teo *et al* (2002) describing a different vascular density and phenotype in pure DCIS *vs* DCIS associated with invasive carcinoma, the latter showing significantly greater numbers of CD34+ and CD141+ vessels and fewer staining for FVIII (Teo *et al*, 2002). Besides distinct vascular profiles in pure and coexistent DCIS, that study also showed a significant negative correlation between vascular density and the extent of necrosis of the tumour, and a correlation between vascular density and the nuclear grade was noted, being highest in the intermediate grade DCIS (Teo *et al*, 2002). The latter is also in agreement with our observation that the association of the proangiogenic growth factors and receptors with *pure* DCIS was considerably more pronounced in the subgroup of non-high-grade DCIS as compared with high-grade DCIS. This could indicate that the group of non-high-grade DCIS without coexistent invasive carcinoma is particularly angiogenic. Our results on different expression patterns of angiogenic factors in non-high-grade as opposed to high-grade DCIS is also compatible with the notion that distinct progression routes exist in the evolution from DCIS to invasive breast cancer (Buerger *et al*, 1999; Mommers *et al*, 2001). Also, the similarity in expression of ET_A_R, VEGF-C, Flt-4, and Her-2/neu between the *in situ* and the concomitant invasive carcinomas and the observed close concordance with respect to nuclear grading between both components is in agreement with previous reports on different lines of genetic evolution of invasive breast cancer (Buerger *et al*, 1999, 2001; Mommers *et al*, 2001).

With respect to our findings, it is conceivable that determination of VEGF-C, ET_A_R, KDR, and Flt-4 expression may facilitate discrimination of a more angiogenic subset within the group of non-high-grade DCIS without coexistent invasive carcinoma. Among non-high-grade DCIS, this more angiogenic group may have a higher probability of recurrence or of progression to invasiveness and thus presumably necessitates a risk-adapted therapy. This is of major clinical importance since approximately half of the local recurrences after treatment for a primary DCIS are invasive and no appropriate tumour marker is available yet that would allow for prediction of recurrence. Moreover, during the last years, antiangiogenic therapy for malignancies has been extensively investigated, and many novel agents representing several approaches to blocking tumour neovascularisation are in clinical trials (Eskens, 2004).

In conclusion, this study showed expression of VEGF-C and its receptor Flt-4, both representing lymphangiogenic growth factors, in intraductal tumour cells of nearly all DCIS lesions. Furthermore, most of the DCIS cases showed significant expression of various angiogenic growth factors. Our data therefore indicate that *in situ* carcinomas are capable of inducing angiogenesis and lymphangiogenesis. Moreover, we found a higher angiogenic activity in DCIS *without* coexistent invasive carcinoma (*pure* DCIS) as compared to DCIS *with* concomitant invasive carcinoma. This association of angiogenic factors with pure DCIS was considerably more pronounced in the subgroup of non-high-grade DCIS as compared with high-grade DCIS. One can therefore assume from these data that the group of non-high-grade DCIS without coexistent invasive carcinoma is particularly angiogenic. Determination of these angiogenic markers in pure DCIS may therefore facilitate discrimination of a more angiogenic subset with a potentially higher risk of progression. This subset of patients could benefit from a risk-oriented, targeted antiangiogenic therapy which represents a potential strategy for prevention of progression of DCIS to invasion.

## Figures and Tables

**Table 1:** List of antibodies and staining procedures used

	**Species**	**Pretreatment**	**Dilution**	**Incubation times**	**Detection**	**Pab source**	**Positive control**
*Growth factors*							
VEGF-A	Rabbit Poly	PK (10 min)	1:15	25 min PAb, 20 min Link AP, 20 min Strept AP, 2^*^8 min modified ‘Neufuchsin’	LSAB/AP	Santa Cruz	Inflammatory tissue
VEGF-C	Rabbit Poly	S (35 min)	1:25	25 min PAb, 20 min Link AP, 20 min Strept AP, 2^*^8 min modified ‘Neufuchsin’	LSAB/AP	Santa Cruz	Inflammatory tissue
bFGF	Rabbit Poly	MW 2 × 7 min (450 W)	1:2000	30 min Dako Blocking Solution, 210 min PAb at 37°C, 30 min Bridge-Ab at 23°C, 30 min 2P^ndP^ Bridge-Ab at 23°C	APAAP	Santa Cruz	Inflammatory tissue
ET-1	Mouse MAb	S (35 min)	1:500	25 min PAb, 20 min Link AP, 20 min Strept AP, 2^*^8 min DAB	LSAB/HRP	Affinity BioReagents	Ovarian carcinoma
							
*Receptors*							
Flt-1 (VEGFR-1)	Rabbit Poly	MW 2 × 7 min (450 W)	1:400	30 min Dako Blocking Solution, 210 min PAb at 37°C, 30 min Bridge-Ab at 23°C, 30 min 2P^ndP^ Bridge-Ab at 23°C	APAAP	Santa Cruz	Inflammatory tissue
KDR (VEGFR-2)	Mouse MAb	MW 2 × 7 min (450 W)	1:400	30 min Dako Blocking Solution, 210 min PAb at 37°C, 30 min Bridge-Ab at 23°C, 30 min 2P^ndP^ Bridge-Ab at 23°C	APAAP	Santa Cruz	Inflammatory tissue
Flt-4 (VEGFR-3)	Rabbit Poly	S (35 min)	1:400	25 min PAb, 20 min secondary Agent (Link AP), 20 min tertiary Agent (Strept AP), 2^*^8 min modified ‘Neufuchsin’	LSAB/AP	Santa Cruz	Inflammatory tissue
bFGF-R1	Rabbit Poly	MW 2 × 7 min (450 W)	1:400	30 min Dako Blocking Solution, 210 min PAb at 37°C, 30 min Bridge-Ab at 23°C, 30 min 2P^ndP^ Bridge-Ab at 23°C	APAAP	Santa Cruz	Inflammatory tissue
ET_A_R	Sheep MAb	Reveal 5 min; H_2_O 5 min; Aurion BSA (1:30) 30 min	1:800	25 min PAb, 60 min RAS (1 :25) at 23°C, 60 min Envision Detection Kit at 23°C	LSAB/HRP	Alexis	Prostate carcinoma
ET_B_R	Sheep MAb	S (35 min)	1:100	25 min PAb, 20 min secondary Ab (Rabbit-Anti-Sheep), 20 min Link AP, 20 min Strept AP, 2^*^8 min DAB	LSAB/HRP	Alexis	Smooth muscle tissue

bFGF=basic fibroblast growth factor; VEGF=vascular endothelial growth factor; ET=endothelin; R=receptor; Flt=fms-like tyrosine kinase; KDR=kinase domain receptor; MAb=monoclonal antibody; Poly=polyclonal antibody; PAb=primary antibody; MW=microwave; Citr.=citrate (pH 6.0); WB=waterbath; S=steamer; PK=proteinase k; o/n=overnight; AP=alcalic phosphatase; Strept=labelled streptavidin; DAB=3,3-diaminbenzidine; LSAB=labelled streptavidin biotin; HRP=horseradish peroxidase; APAAP=alcalic phosphatase anti-alcalic phosphatase. Santa Cruz Biotechnology Inc., Santa Cruz, USA. Affinity BioReagents, Golden, CO, USA. Alexis, Lausen, Switzerland.

**Table 2:** Expression of growth factors and their receptors in tumour cells of DCIS (n (%) of evaluable cases with positive immunoreaction)

**Antigen**	**Total**	**Pure DCIS**	**Coexistent DCIS**	***P* a**
Growth factors				
VEGF-A	80/175 (45.7%)	40/88 (45.5%)	40/87 (46%)	NS
VEGF-C	152/172 (88.4%)	78/80 (97.5%)	74/92 (80.4%)	*P*<0.001
bFGF	20/163 (12.3%)	11/78 (14.1%)	9/85 (10.6%)	NS
ET-1	78/161 (48.4%)	32/75 (42.7%)	46/86 (53.5%)	NS
				
*Growth factor receptors*				
VEGFR-1 (Flt-1)	27/168 (16.1%)	2/79 (2.5%)	25/89 (28.1%)	*P*<0.001
VEGFR-2 (KDR)	84/157 (53.5%)	47/75 (62.7%)	37/82 (45.1%)	*P*=0.028
VEGFR-3 (Flt-4)	165/173 (95.4%)	81/82 (98.8%)	84/91 (92.3%)	*P*=0.043
bFGF-R1	151/160 (94.4%)	77/80 (96.3%)	74/80 (92.5%)	NS
ET_A_R	126/165 (76.4%)	69/81 (85.2%)	57/84 (67.9%)	*P*=0.009
ET_B_R	60/159 (37.7%)	28/79 (35.4%)	32/80 (40%)	NS

NS=nonsignificant.

a *χ*^2^ test (pure DCIS *versus* coexistent DCIS).

**Table 3:** Coexpression of ligand and respective receptor in the same DCIS specimen indicating potential autocrine loops

**Ligand/Receptor**	**n/total (%)**
VEGF-A/Flt-1	13/166 (7.8%)
VEGF-A/KDR	36/147 (24.5%)
	
VEGF-C/Flt-4	143/165 (86.7%)
VEGF-C/KDR	78/152 (51.3%)
	
bFGF/bFGF-R1	17/163 (10.4%)
	
ET-1/ET_A_R	60/161 (37.3%)
ET-1/ET_B_R	42/161 (26.1%)

**Table 4:** Expression of growth factors and growth factor receptors stratified for nuclear grading

**Factor**	**Nuclear grade**	**Pure DCIS**	**Coexistent DCIS**	***P* (*χ*^2^ test)**
*Growth factorsU*				
VEGF-A	Non-high-grade^a^	25/55 (45.5%)	17/35 (48.6%)	NS
	High-grade	15/33 (45.5%)	21/49 (42.9%)	NS
				
VEGF-C	Non-high-grade	50/50 (100%)	27/37 (73%)	*P*<0.001
	High-grade	28/30 (93.3%)	44/52 (84.6%)	NS
				
bFGF^b^	Non-high-grade	10/51 (19.6%)	6/35 (17.1%)	NS
	High-grade	1/27 (3.7%)	2/47 (4.3%)	NS
				
ET-1	Non-high-grade	21/48 (43.8%)	16/34 (47.1%)	NS
	High-grade	11/27 (40.7%)	29/49 (59.2%)	NS
				
*Receptors*				
				
Flt-1 (VEGFR-1)	Non-high-grade	1/50 (2%)	13/36 (36.1%)	*P*<0.001
	High-grade	1/29 (3.4%)	12/50 (24%)	*P*=0.018
				
KDR (VEGFR-2)	Non-high-grade	33/50 (66%)	12/34 (35.3%)	*P*=0.006
	High-grade	14/25 (56%)	23/45 (51.1%)	NS
				
Flt-4 (VEGFR-3)	Non-high-grade	50/50 (100%)	33/37 (89.2%)	*P*=0.017
	High-grade	31/32 (96.9%)	48/51 (94.1%)	NS
				
bFGF-R1	Non-high-grade	47/49 (95.9%)	29/32 (90.6%)	NS
	High-grade	30/31 (96.8%)	43/46 (93.5%)	NS
				
ET_A_R	Non-high-grade	44/53 (83%)	22/36 (61.1%)	*P*=0.020
	High-grade	25/28 (89.3%)	32/45 (71.1%)	NS
				
ET_B_R	Non-high-grade	20/49 (40.8%)	12/32 (37.5%)	NS
	High-grade	8/30 (26.7%)	18/45 (40%)	NS

NS=nonsignificant.

a Non-high-grade=low + intermediate grade DCIS.

b Logistic regression showed a significant correlation between bFGF expression and grading (*P*=0.009).

**Table 5:** Distribution of clinical and histopathological characteristics in the subgroups of pure *vs* coexistent DCIS

**Parameter**	**Pure DCIS**	**Coexistent DCIS**	***P***
Patients' age (years)	60.5±13.6	57.3±14.4	NS
			
*Estrogen receptor*			
Negative	46/70 (65.7%)	57/78 (73.1%)	NS
Positive	24/70 (34.3%)	21/78 (26.9%)	
			
*Progesterone receptor*			
Negative	41/69 (59.4%)	50/77 (64.9%)	NS
Positive	28/69 (40.6%)	27/77 (35.1%)	
			
*Her-2/neu*			
Negative	57/73 (78.1%)	57/73 (78.1%)	NS
Positive	16/73 (21.9%)	16/73 (21.9%)	
			
*Nuclear grade*			
Non-high-grade	57/96 (59.4%)	46/101 (45.5%)	NS
High-grade	39/96 (40.6%)	55/101 (54.5%)	

NS=nonsignificant.
